# Use of the QuantiFERON-TB Gold In-Tube Test in the Diagnosis and Monitoring of Treatment Efficacy in Active Pulmonary Tuberculosis

**DOI:** 10.3390/ijerph14030236

**Published:** 2017-02-27

**Authors:** Ping-Chin Chang, Pin-Hui Wang, Kow-Tong Chen

**Affiliations:** 1 Division of Infectious Diseases, Department of Internal Medicine, Chi-Mei Medical Center, Liouying, Tainan 736, Taiwan; 2LH101@TMH.org.tw; 2 Department of Public Health, College of Medicine, National Cheng Kung University, Tainan 701, Taiwan; e8755013@yahoo.com.tw; 3 Department of Occupational Medicine, Tainan Municipal Hospital, Tainan 701, Taiwan

**Keywords:** QuantiFERON-TB Gold In-Tube test, pulmonary tuberculosis, sensitivity, specificity

## Abstract

The value of QuantiFERON in the diagnosis of tuberculosis disease and in the monitoring of the response to anti-tuberculosis treatment is unclear. The aims of this study were to evaluate the accuracy of the QuantiFERON-TB Gold In-Tube (QFT-GIT) test in the diagnosis of tuberculosis and in the monitoring of the response to anti-tuberculosis treatment in patients with active pulmonary tuberculosis (PTB). Between January 2013 and December 2015, 133 cases with active PTB and 133 controls with no mycobacterial infection, matched by age (within 3 years) and by the week that they visited Tainan Chest Hospital, were enrolled in the study. Serial testing by QFT-GIT at baseline and after 2 and 6 months of treatment was performed. At these time points, a comparison of the performance of QFT-GIT with that of sputum culture status among study subjects was conducted. Compared to baseline, 116 (87.2%) cases showed a decreased response, whereas 17 (12.8%) showed persistent or stronger interferon-gamma (IFN-γ) responses at 2 months. PTB patients IFN-γ responses declined significantly from baseline to 2 months (median, 6.32 vs. 4.12; *p* < 0.005). The sensitivity values of the QFT-GIT test for the detection of pulmonary tuberculosis at cut-off points of 0.35 IU/mL, 0.20 IU/mL, and 0.10 IU/mL were 74.4%, 78.2%, and 80.5%, respectively. The specificity values at cut-off points of 0.35 IU/mL, 0.20 IU/mL, and 0.10 IU/mL were 66.2%, 63.9%, and 57.1%, respectively. Our results support the QFT-GIT assay as a potential tool for diagnosing tuberculosis and for monitoring the efficacy of anti-tuberculosis treatment.

## 1. Introduction

Tuberculosis (TB) remains an important global public health problem, with an estimated 2 billion people who are infected, especially in high-burden low-income countries [1,2]. Thus, the early diagnosis of TB and the prevention of reactivation of latent tuberculosis infection (LTBI) are important in controlling the TB epidemic [3]. However, the early detection of infection with *Mycobacterium tuberculosis* (Mtb) remains a complicated issue pertaining to the control and prevention of TB. For many years, the tuberculin skin test (TST) was the test most commonly used for the diagnosis of TB infection due to its low cost and convenience in most countries. However, this method has several disadvantages, including poor specificity in people who received the Bacille Calmette-Guerin (BCG) vaccination or who were infected with non-tuberculous mycobacteria (NTM), low sensitivity in immunocompromised persons, and the requirement of two clinical visits to read the results [4,5]. In the last decade, interferon gamma release assays (IGRAs) have been introduced to aid in the detection of LTBI. IGRAs detect the ex vivo release of the key anti-tuberculosis cytokine interferon-gamma (IFN-γ) [5]. Previous studies have demonstrated that using IGRAs may be an alternative approach for diagnosing TB [6,7]. IGRAs include proteins that are almost exclusively present in Mtb than those in the purified derivative (PPD) and that are encoded by genes located in the region of difference 1 (RD 1) within the Mtb genome. These genes are not found in *M. bovis*, BCG, or most environmental mycobacteria [8]. The QuantiFERON-TB Gold In-Tube test (QFT-GIT) assay (Cellestis, Carniege, Victoria, Australia) measures the IFN-γ concentration in whole blood after stimulation by specific tuberculosis antigens (e.g., early secreted antigenic target-6 (ESAT6), culture filtrate protein-10 (CFP10)), and TB7.7 antigen [9,10]). The QFT-GIT has already been recognized as an efficient alternative test to detect the presence of LTBI [9,10,11,12]; however, whether the QFT-GIT will be useful in monitoring the responses to anti-tuberculosis treatment is unclear [10,13]. The potential prognostic use of IFN-γ responses has been studied in research describing isoniazid (INH) treatment of LTBI and in anti-tuberculosis treatment. Regarding LTBI, the prognostic use of IFN-γ has not yet been clearly established. It has been reported that the IFN-γ responses after INH prophylaxis may be stronger [14], persistent [15], decreased [16], or dependent on the antigen used [17,18]. Similarly, in the treatment of active TB, some studies have observed post-treatment mitigation of the IFN-γ response [19,20,21], while others have reported persistent or even stronger IFN-γ responses after anti-tuberculosis treatment [22,23,24,25]. Several studies on the performance of the QuantiFERON test have been conducted in Taiwan [26,27,28,29,30]; however, these studies were confined to the diagnosis efficacy of LTBI and the comparison between the QuantiFERON test and TST. The aim of this study was to assess the potential use of the QFT-GIT test in the diagnosis, monitoring efficacy, and treatment of individuals with and without active TB, and to evaluate the factors that influenced the performance of the test.

## 2. Materials and Methods

### 2.1. Setting

This study was conducted at the Tainan Chest Hospital of the Ministry of Welfare and Health in Taiwan. The Tainan Chest Hospital provides respiratory disease services such as voluntary counseling and testing of tuberculosis, medical care, and laboratory testing. More than 4320 people with respiratory disorders visit this hospital each year. Of these people, 300 (7%) were diagnosed with tuberculosis.

### 2.2. Study Population

This was a case-control study. Cases were identified from medical records of patients presenting with active pulmonary tuberculosis (PTB) at the Chest Hospital of the Ministry of Welfare and Health between January 2013 and December 2015. Active PTB cases were defined as patients with a history of cough for more than 3 weeks and with positive cultures for Mtb complex in ≥1 sputum sample [31]. If both *M. tuberculosis* complex and NTM were identified, we classified the subjects as having active PTB. Participants were classified as NTM culture positive when NTM was isolated in ≥1 sputum sample. The remaining participants were classified as having no mycobacterial infection. In this study, we assessed the clinical characteristics and outcomes among cases with active PTB when compared with those participants classified as having no mycobacterial infection.

The exclusion criteria were age <18 years, pregnancy, positive human immunodeficiency virus (HIV) serology, immunosuppressive drug use, no data from the QFT-GIT test, and a previous anti-tuberculosis treatment history. Informed consent was obtained before sample collection. Patients who agreed to participate in the study and who were willing to attend regular follow-up visits were asked to complete a questionnaire that included limited demographic information. Participants provided first spot and second early morning sputum specimens for the laboratory study of *M. tuberculosis*. Laboratory testing of the sputum was performed by the Laboratory Division of the Chest Hospital.

One control was selected for each case patient. All controls were patients visiting Chest Hospital presenting with respiratory-related illness without mycobacteria in their sputum culture, and they were matched to case patients in terms of visiting the Chest Hospital in the same week, sex, and by age (within three years). Controls who were pregnant, had positive HIV serology, immunosuppressive drug use, or no data for the QFT-GIT test were excluded.

### 2.3. Study Procedures

All the study patients underwent QFT-GIT assay, chest X-ray examination, and sputum culture at baseline and after 2 months. All patients who were enrolled in the study received a standardized anti-tuberculosis treatment, including daily INH, rifampicin (RMP), ethambutol (EMB), and pyrazinamide (PZA), for 2 months, with a follow-up at the end of 2 months of treatment. Patients who remained sputum culture positive at the end of 2 months were given INH, RMP, PZA, and EMB for an additional month; if they remained culture positive at the end of 3 months, they were excluded from the study and treated according to their culture and drug sensitivity patterns.

Review and approval for the study were obtained from the Institutional Review Board for the Protection of Human Subjects at Tainan Chest Hospital and National Cheng Kung University Hospital, Taiwan (ER-99-343).

### 2.4. Laboratory Tests

Sputum samples were decontaminated and centrifuged, and the pellet was used to prepare smears that were examined microscopically and then graded for acid-fast bacilli using the Ziehl-Neelsen technique and fluorescence microscopy following auramine O staining. Decontaminated specimens were inoculated in BACTEC 7H9 liquid medium for the isolation and identification of *M. tuberculosis*. The QFT-GIT test was conducted on 1 mL of venous blood that was incubated at 37 °C for 16–24 h. These procedures have been described elsewhere [28,29]. Calculations were performed using the QFT-GIT analysis software provided by the manufacturer. QFT-GIT was considered positive if the estimated IFN-γ concentration in the sample exceeded the negative control by 0.35 international units (IU)/mL. All IFN-γ concentration values in subsequent analyses represent the difference between the samples and their negative controls.

Additional specimens from 10 participants were sent to the Taiwan Centers for Disease Control (Taiwan CDC) for laboratory test replication (QFT); the results from the Taiwan CDC and Tainan Municipal Hospital were in good agreement.

### 2.5. Statistical Analysis

Baseline patient characteristics were expressed as medians (ranges) and inter-quartile ranges (IQRs) (continuous variables) and as numbers and percentages (categorical variables). The prognostic accuracy of QFT-GIT was examined using the parameters of a diagnostic test: sensitivity, specificity, positive predictive value (PPV), and negative predictive value (NPV). Moreover, the Receiver Operating Characteristic (ROC, plots the sensitivity to 1 minus specificity) for the prognostic value of the QFT-GIT assay at baseline, to predict the likelihood of sputum culture positive at 2 months, was included and diagnostic accuracy was assessed by calculating the areas under the ROC curves [32]. Chi-square tests, Fisher’s exact tests, and Student’s *t*-tests were used to analyze the differences in the distribution of study subjects by IFN-γ response (failing vs. persistent or stronger) at 2 months and at 6 months. The significance of the derived *p*-values was defined as an alpha level of 0.05 or less. All the analyses were conducted using Stata 9.0 (Stata Corp, College Station, TX, USA).

### 2.6. Definition

Sensitivity was defined as the proportion of people with the disease who had a positive test for the disease. Specificity was defined as the proportion of people without the disease who had a negative test for the disease. The positive predictive value (PPV) was defined as the probability of disease in a patient with a positive test result. The negative predictive value (NPV) was defined as the probability of not having the disease when the test result was negative. The predictive value was determined by the sensitivity and specificity of the test and by the prevalence of disease in the population being tested. The predictive value is regarded as the most relevant characteristic in the clinician’s interpretation of the test results [5].

## 3. Results

Table 1 displays the demographic characteristics of the study subjects. During the study period, a total of 300 cases were identified as eligible study subjects. Of these subjects, 266 (88.7%) met the inclusion criteria and were enrolled in the study. The mean age of the study subjects was 58.6 years (inter-quartile range (IQR): 49.6–71.2 years); 180 (67.7%) were male, and 162 (60.9%) were considered positive at baseline by QFT-GIT testing. Compared with controls, the cases had higher rates of QFT-GIT test positivity (*p* = 0.001) and higher levels of IFN-γ (*p* < 0.001). Sex and age were not significantly different between the cases and controls (*p* > 0.05 in all).

**Figure 1:**
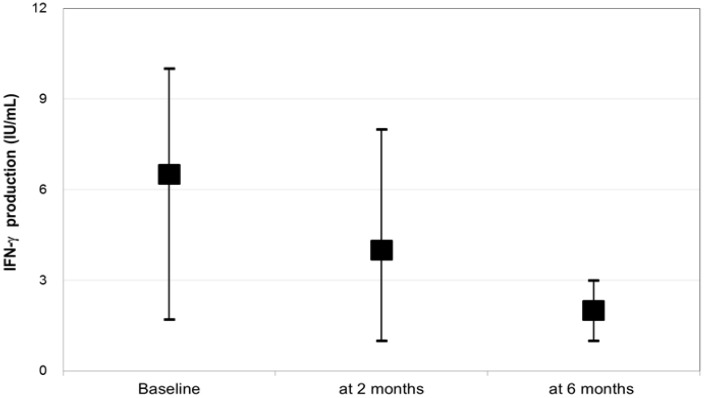
IFN-γ production levels were determined using serial QFT-GIT assays (at baseline, 2 months, and 6 months after treatment initiation) in subjects with active tuberculosis who were on a standard regimen (*N* = 133).

Figure 1 shows the production level of IFN-γ at baseline, 2 months, and 6 months at the cut-off point of 0.35 IU/mL. IFN-γ responses declined significantly from baseline to 2 months (median, 6.32 IU/mL vs. 4.12 IU/mL; *p* < 0.001) and again from 2 months to 6 months (median, 4.12 IU/mL vs. 2.04 IU/mL; *p* < 0.001). Compared to the baseline, 116 (87.2%) cases had a decreased response, and 17 (12.8%) had persistent interferon-gamma (IFN-γ) responses at 2 months after having positive test cultures and at the end of an intensive phase of anti-tuberculosis treatment. Eight (6%) cases remained culture positive at 6 months post-treatment.

### Accuracy of the QFT-GIT Test

Table 2 displays the diagnostic accuracy of the QFT-GIT test for the detection of active pulmonary tuberculosis at different cut-off values. At a QFT-GIT cut-off value of ≥0.35 IU/mL, the sensitivity was 74.4%, the specificity was 66.2%, the PPV was 68.8%, and the NPV was 72.2% for the QFT-GIT test for PTB. At a QFT-GIT cut-off value of ≥0.20 IU/mL, the sensitivity was 78.2%, specificity was 63.9%, PPV was 68.4%, and the NPV was 74.6% for the QFT-GIT test for PTB. At a QFT-GIT cut-off value of ≥0.10 IU/mL, the sensitivity was 80.5%, specificity was 57.1%, PPV was 65.2%, and the NPV was 74.5% for the QFT-GIT test for PTB. 

**Figure 2:**
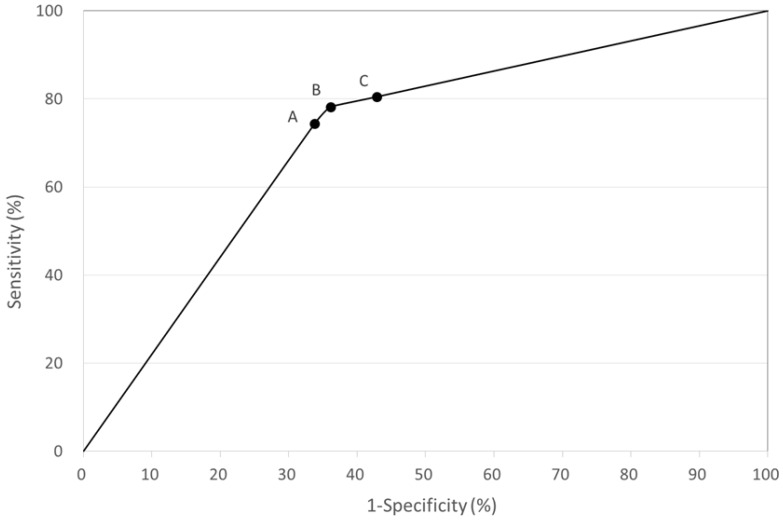
A Receiver Operator Characteristics (ROC) curve for the prognostic value of QFT-GIT assay at baseline to predict the likelihood of sputum culture positive at 2 months. A: QFT-GIT cut-off point at ≥0.35 IU/mL; B: cut-off point at ≥0.20 IU/mL; C: cut-off point at ≥0.10 IU/mL.

Figure 2 shows the ROC curve. The prognostic performance of QFT-GIT at baseline was maximal at the cut-off value of ≥0.20 IU/mL. This information translated to a prognostic odds ratio (OR) of 6.35 (3.69–10.93), suggesting that subjects with QFT-GIT measured IFN-γ responses ≥0.20 IU/mL had over 6.35 times higher risk of being culture-positive at 2 months.

## 4. Discussion 

Few studies have evaluated the diagnostic and prognostic value of the QIT-GIT for PTB in Taiwan. Chen et al. [30] reported that IFN-γ responses declined significantly from baseline to 2 months and that the sensitivity of the QFT-GIT test for the detection of pulmonary tuberculosis at a cut-off point of 0.35 IU/mL was 71.4%, with a specificity of 64.3%. This study provides important information regarding the role of QFT-GIT assays in the monitoring of active PTB treatment. However, the number of patients included in the study was small.

Except for studies from Japan [11], Korea [33], and India [34], most other published studies [35,36,37,38,39] have reported that the QFT-GIT assay has moderate sensitivity (61%–81%). In this study, we had similar findings, with the QFT-GIT assay having a sensitivity of 74.4% and specificity of 66.2% for active PTB detection. These findings are important, as the accuracy of IFN-γ responses has not been unequivocally established for the diagnosis of active TB. Previous studies [5,40] showed that the QFT-GIT assay performed better than the TST for the diagnosis of tuberculosis. However, neither test is reliable for the diagnosis of TB.

Serial testing by QFT-GIT demonstrated an overall progressive weakening of the IFN-γ response during anti-tuberculosis treatment, and QFT-GIT assessment after 2 months of treatment could be an independent and sensitive indicator of the likelihood of failing to convert sputum culture status. Our study showed that 11% of study subjects had persistent IFN-γ at 2 months and were culture positive at the end of the anti-tuberculosis treatment. A previous study [34] suggested that nearly half of the study cohort was still positive as determined by QFT-GIT after 6 months of anti-tuberculosis treatment. In this study, 6% (8 cases) were still positive at follow-up after 6 months of anti-tuberculosis treatment. There are several possible explanations for why immune responses to even specific antigens (ESAT6 and CFP10) may not have dropped below predefined levels and resulted in positive tests after anti-tuberculosis treatment: (1) T-cell responses to ESAT6 may persist as an after effect of previously treated or quiescent infection [29]; (2) the anti-tuberculosis treatment may only have helped the infection revert to a stage of latency rather than conferring sterilizing immunity [41]; (3) it has been argued that in some individuals, a population of activated T-cells persists in the absence of direct mycobacterial antigen stimulation, even for several years after completing treatment [30]; (4) it is possible that continued exposure to *M. tuberculosis* during anti-tuberculosis treatment could produce a continuous immune response, especially as the environmental burden is high; and (5) there is inter-individual variation in the strength of the IFN-γ response that can be partially explained by genetic polymorphisms in the host [42]. Although the IFN-γ level measured by the QFT-GIT assay decreased after successful anti-TB treatment in most patients, many of the patients exhibited QFT-GIT reversion to negativity. Thus, the reversion to negativity of the QFT-GIT assay may not be a good surrogate for the treatment response. Of course, the short follow-up time could have affected these results.

Our study found that the accuracy of the QFT-GIT assay varied according to the cut-off point. A cut-off point of 0.35 IU/mL for the diagnosis of active TB had moderate sensitivity (74.4%) and specificity (66.2%). If the cut-off point was set at 0.20 IU/L, the sensitivity increased to 78.2%, but the specificity decreased to 63.6%. Similarly, if the cut-off point was at 0.10 IU/mL, the sensitivity increased to 80.5%; however, the specificity was 57.1%. Consequently, when using the QFT-GIT assay for monitoring the response to treatment, it may be necessary to revise the cut-off to be prognostically meaningful [34]. Future studies will need to address this issue more directly using larger numbers of patients treated for active TB.

The QuantiFERON TB Plus includes an additional antigen tube to QFT-GIT, which contains ESAT6/CEP10 peptides that are intended to specifically induce a CD8+ T-cell lymphocytes response in addition to the CD4+ T-cell response detected with the original QFT-GIT assay [43]. The new CD8+ -specific peptides have been added to increase the sensitivity of the test because it has been shown that Mtb-specific CD8+ T-cells are mainly associated to active TB [44,45,46,47]. The first data on the performance of QuantiFERON TB Plus were reported recently [48,49,50]. When compared with the previous test version of QFT-GIT [50], equal sensitivity of the new generation QFT-plus was observed. However, at the moment it is unknown the performance of the QFT-plus as a tool to monitor TB therapy.

There were several limitations in our study. First, TST status may influence the QFT-GIT results [51,52]. In our study, we did not evaluate the influence of TST status on the prognostic performance of QFT-GIT. Second, our study used a mycobacterial culture as the gold standard for the diagnosis of TB. This methodology sometimes gives false negative results due to poor sputum sample collection or paucibacillary sputum samples [53]. Despite these limitations, our results support the utilization of the QFT-GIT assay as a potential tool to monitor the efficacy of anti-tuberculosis treatment in cases of active PTB.

## 5. Conclusions 

This study may underestimate the performance of the QFT-GIT test for the diagnosis of TB. Our study indicates that QFT-GIT has moderate sensitivity and specificity; our results support the candidacy of the QFT-GIT assay as a potential tool for the diagnosis of tuberculosis and for monitoring the efficacy of anti-tuberculosis treatment. 

## Figures and Tables

**Table 1:** Demographic characteristics of the study subjects in Chest Hospital, Taiwan.

Variables	Total *N* = 266	Cases *n* = 133	Controls *n* = 133	*p* Value *
Age (year) (median) (IQR)	58.6 (49.6–71.2)	56.2 (47.3–70.4)	62.3 (51.2–72.1)	0.17
Sex				0.19
Male (%)	180 (68)	85 (64)	95 (71)	
Female (%)	86 (32)	48 (36)	38 (29)	
QFT-GIT test				0.001
Positive (%)	162 (61)	95 (71)	48 (36)	
Negative (%)	104 (39)	38 (29)	85 (64)	
Median IFN-γ response (IU/mL) (IQR)	3.54 (0.51–10.0)	6.32 (1.01–10.0)	0.76 (0.01–10.0)	<0.001

* comparison of cases to controls; QFT-GIT: QuantiFERON-TB Gold In-Tube; IFN-γ: interferon-gamma; IQR: inter-quartile range; IU: international units.

**Table 2:** Diagnostic accuracy of the QFT-GIT test in the detection of active PTB.

QFT-GIT Test Cut-Off Value (IU/mL)	Sensitivity (%)	Specificity (%)	PPV (%)	NPV (%)
≥0.35	74.4	66.2	68.8	72.1
≥0.20	78.2	63.6	68.4	74.6
≥0.10	80.5	57.1	65.2	74.5

PTB: pulmonary tuberculosis; PPV: positive predictive value; NPV: negative predictive value.
